# Enhanced Child Care: Contrast Correction for Pediatric Hip Ultrasound Using Hyperanalytic Wavelets

**DOI:** 10.3390/jpm12081328

**Published:** 2022-08-18

**Authors:** Beatrice Arvinti, Emil Radu Iacob, Alexandru Isar, Daniela Iacob, Marius Costache

**Affiliations:** 1 Fundamentals of Physics for Engineers Department, “Politehnica” University Timisoara, Bd. Vasile Pârvan 2, 300223 Timisoara, Romania; 2 Department of Pediatric Surgery, “Victor Babes” University of Medicine and Pharmacy, Eftimie Murgu Square 2, 300041 Timisoara, Romania; 3 Faculty of Electronics, Telecommunications and Information Technologies, “Politehnica” University Timisoara, Bd. Vasile Pârvan 2, 300223 Timisoara, Romania; 4 Department of Neonatology, “Victor Babes” University of Medicine and Pharmacy, Eftimie Murgu Square 2, 300041 Timisoara, Romania

**Keywords:** neonates, hip ultrasound, image filtering, speckle noise, wavelet analysis

## Abstract

(1) Background: The prevention of critical situations is a key ability in medicine. Hip ultrasound for neonates is a standard procedure to prevent later critical outcomes, such as hip dysplasia. Additionally, the SARS-CoV-2 pandemic has put worldwide stress upon healthcare units, resulting often in a lack of sufficient medical personnel. This work aims to develop solutions to ease and speed up the process of coming to a correct diagnosis. (2) Methods: Traditional medical procedures are envisaged, but they are enhanced to reduce diagnosing errors due to the movements of the neonates. Echographic noise filtering and contrast correction methods are implemented the Hyperanalytic Wavelet Transform, combined with an adaptive Soft Thresholding Filter. The algorithm is tailored to infants’ structure and is tested on real ultrasounds provided by the “Victor Babes” University of Medicine and Pharmacy. Denoising and contrast correction problems are targeted. (3) Results: In available clinical cases, the noise affecting the image was reduced and the contrast was enhanced. (4) Discussion: We noticed that a significant amount of noise can be added to the image, as the patients are neonates and can hardly avoid movements. (5) Conclusions: The algorithm is personalized with no fixed reference value. Any device easing the clinical procedures of physicians has a practical medical application.

## 1. Introduction

**Figure 1:**
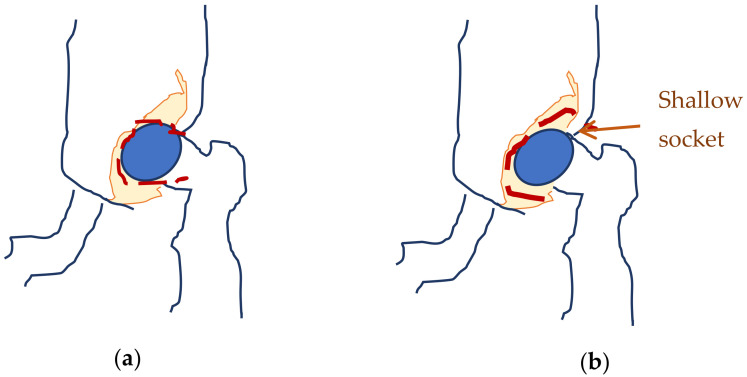
Normal hip (**a**) and hip dysplasia (**b**) in neonates.

Neonates are prone to affections, which typically do not produce visible effects while the infants are less than one year old, due to their reduced movements. Hip dysplasia is one of the most occurring affections [1]. Developmental hip dysplasia (also known as congenital dislocation of the hip) is an abnormality of the hip joint—the articulation between the head of the femur and the acetabulum of the pelvis. It results in an increased risk for joint dislocation, as the socket portion (acetabulum) does not fully cover the ball portion (head of the femur), as can be observed in Figure 1 [2] The incidence of this disease varies between 1.5–25/1000 live births [3]. It can occur at birth, or develop later during growth, and occasionally one leg may be shorter than the other [4], which would hinder the child’s natural growth. Usually, the anterior section of the acetabular cartilage is the most vulnerable to the tearing of the acetabula [5]. Without treatment, complications in neonates may occur, such as limping, arthritis, and low back pain [6]. Additionally, the elderly are prone to fractures of the thin neck, which attaches the femur to the shaft, mostly due to degenerative effects, such as those induced by osteoporosis [7].

A pediatric hip exam was carried out with the help of an ultrasound machine [8,9], which examined the hip’s joints, searching for possible defects. This imaging technique indicates the location of the femoral head in relation to the acetabulum and can measure specific parameters, such as the inclination of the acetabular roof or the depth of the acetabulum [10]. Usually, the process is tiresome for the neonate as it takes around 15 min, the child must stand still to obtain good resolutions and both hips must be scanned for comparison.

In any ultrasound exam, a transducer is pressed against the skin and sends high-frequency sound waves inside the body-tissue, cartilages, and bones, all of which react differently (different density), aiming to form images of the hip joint [8].

Usually, problems appear when the child moves and the images are not sharp enough. Software filters should be applied for a reliable diagnosis. [11,12,13].

For the ultrasound device some elements are compulsory:

- a linear probe/transducer in the frequency range 5–7 MHz. Probes/transducers with a higher frequency than 7 MHz, show a better resolution but worse penetration depths, thus worse ultrasound images. However, neonates smaller than 4 weeks of age must be examined with the 7 MHz transducer, while in children over this age a 5 MHz transducer is used;
- there must be a possibility of rotating electronically the image (by 90°) on the screen to visualize the “standard projection”;
- there must be a thermal printer available for direct image printing or the possibility of storing the images without loss of resolution;
- the ultrasound device must be performant enough to possess software, which should allow line-tracing to measure the angles formed and thus provide a clinical diagnosis.

The clinical exam relies on a single coronal image of the hip. The child is placed in a lateral decubitus position and the transducer is brought parallel to the studied hip and parallel to the vertebral spine. This is aimed at obtaining a coronal/frontal through the middle plane of the acetabulum (hip socket) [14].

**Figure 2:**
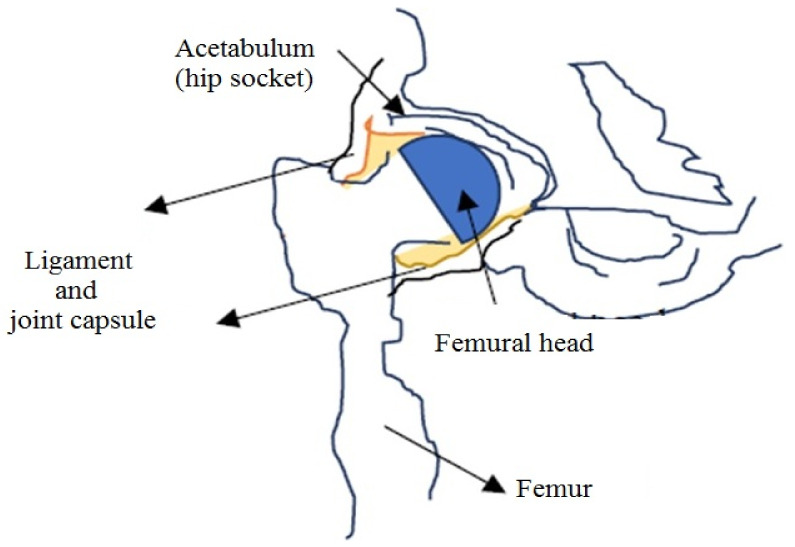
Biological construction of the hip—important for ultrasound measurements.

This “standard plane” is identified through the presence of three points (Figure 2):

- the inferior side of the ilium bones of the pelvis, inside the acetabulum fossa [14];
- the middle plane of the hip socket;
- the acetabular labrum (a cartilage ring that surrounds the acetabulum);

The paper is structured as follows: in Section 1 we present the general overview and necessity of supervising hip dysplasia at neonates. The second section summarizes the Hyperanalytic Wavelet Transform (HWT) and the Adaptive Soft Thresholding Filter (ASTF). Section 3 is devoted to the experimental results obtained by applying the proposed denoising system on real sonographic images. The last section contains the conclusion of the paper and possible directions for further research.

## 2. Materials and Methods

Several noisy images from neonates were checked, the images were acquired in Timisoara, Romania. The proposed filtering algorithm based on the hyperanalytical wavelet has been tested on several newborn hip-images, both male and female patients. Ultrasound images that were not correctly acquired were excluded from the study, as well as images of neonates with known musculoskeletal malformations or pathologies. For each noisy ultrasound image, two clinical aspects (general view of the scanned area and zoom on area of interest) were checked.

During measurements, the physician focused on two coronal angles (acetabular inlet planes) “alpha” and “beta”, some computers displaying a software for the calculation of the Graf angles. Of course, the lines defining the angles must be correctly identified and tracked by the physician [15]:

- an initial line is traced parallel with the lateral wall/side of the ilium bone (constituting the baseline);
- a second line is traced in the middle of the bony margin (with the fibrocartilaginous margin) surrounding the acetabulum, forming the angle “beta” with the baseline: this is a measure of the cartilaginous covering of the acetabulum;
- a third line is traced from the inferior side of the bony margin of the acetabulum to the triradiate cartilage (the Y-shaped plate at the junction where the ischium, ilium, and pubis meet in the skeletally immature skeleton of the neonate), tangentially to the bony margin of the acetabulum. The angle formed with the baseline is the “alpha” angle: this is a measure of the bony covering of the acetabulum. It is usually greater than 60°. An angle less than 55° is considered not normal.

Wavelets are an adaptive, powerful, but easy-to-implement mathematical tool which allows the construction of effective denoising filters. During propagation, the waves associated with the input signals are reflected by obstacles, creating echoes. The ultrasound is generated by measuring the strength and the round-trip time of the received echoes [10]. As the patient is stirring, noise interferes with the useful image. The main noise affecting the sonographic signal is called speckle noise.

**Figure 3:**
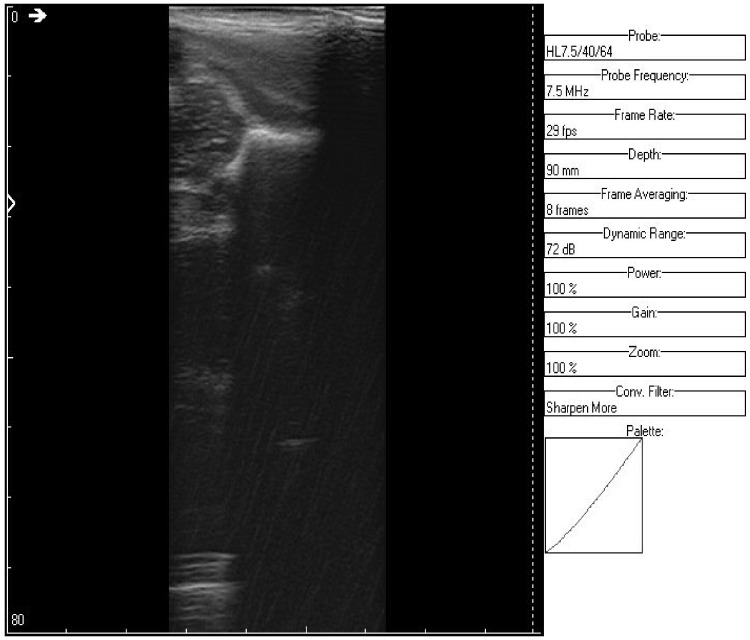
Original recorded data.

Speckle noise affects the mean grey level of an area, causing a difficult image interpretation [16]. It is modeled as a multiplicative noise and is thus harder to filter out. Speckle noise in ultrasound biomedical images is a random granular pattern produced mainly by multiplicative noise that degrades the visual evaluation in ultrasound imaging. It does not correspond to the actual tissue microstructure and tends to mask the presence of low-contrast lesions and fine structures as shown in Figure 3. It is generated by the fact that there are a number of elementary scatters within each resolution cell of the image that reflect the incident wave back towards the ultrasound sensor. The backscattered coherent waves with different phases undergo constructive and destructive interferences in a random manner.

Removing random noise from the original image is still a challenging research in image processing. The presence of speckle noise severely degrades the signal-to-noise ratio (SNR) and contrast resolution of the image, making human interpretation and computer assisted detection techniques difficult and inconsistent. Therefore, a speckle reduction process is quite necessary in low SNR, low contrast ultrasound images for enhancing the visualization of organ anatomy and improving the accuracy of object detection without affecting the important diagnostic features of the image.

In this paper, we aim to remove noise induced by unwanted echoes, which affects the main grey levels of the area, making medical interpretation and diagnosis difficult. A shorter exposition time and a faster diagnosis are also beneficial for the neonate. We propose a novel wavelet transform, the Hyperanalytic Wavelet Transform (HWT) [17,18], associated with an Adaptive Soft Thresholding Filter (ASFT) [19]. This filter represents an optimal solution for the denoising of images affected by additive noise problem [20]. It is a Maximum a Posteriori (MAP) filter equivalent with the Least Absolute Shrinkage and Selection Operator (LASSO). We regard the denoising problem as a regularization problem. The additive noise randomly affects the intensity values of each pixel in a noisy image. Hence, by regularizing the pixel intensity values, we can remove the noise. There has been a tremendous amount of work where researchers spanning a wide range of disciplines have studied the regularization methods. One of these methods is the LASSO-based regularization [21], which is proved to be the equivalence of the ASTF and the LASSO regulator. As the noise induced by tissue reflections is multiplicative, it is quite difficult to design effective filtering algorithms. This is why we apply first a homomorphic procedure (computing the natural logarithm of the acquired image), followed by the HWT and ASTF computations and the logarithm can finally be inverted.

**Figure 4:**
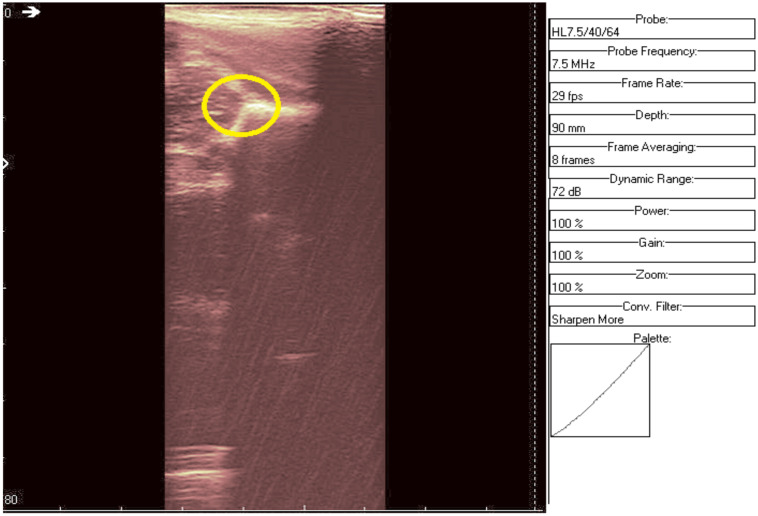
Original data with an added color palette. A region of interest containing some speckle grains was encircled in yellow.

The HWT is used for decomposing the signal on different levels to check different frequencies (layers) of the same input signal, a filter being applied at each level. Finally, the signal is recomposed after denoising (Figure 4). Each wavelet transform uses a bivariate mother wavelet MW $\psi_{a}$(x,y), which is a hyperanalytic function (Equation (1)), to obtain a quasi-shift invariant wavelet transform with enhanced directional selectivity. Additionally, the HWT is implemented with the aid of the univariate Hilbert transform, obtaining complex wavelet coefficients of the ASTF’s input data, which allow more efficient filtering of the image than other wavelet-based denoising methods.

(1) $$\psi_{a}(x,y)=1\psi (x,y)+iH_{x}\{\psi (x,y)\}+jH_{y}\{\psi (x,y)\}+kH_{x}\{H_{y}\{\psi (x,y)\}\}$$

where $\psi_{a}\left(x,y\right)$ is the hypercomplex mother wavelet associated to the signal on each scale and ij = ji = k, jk = kj = i, ki = −ik = j, ik = ki = −j, i^2^ = j^2^ = −k^2^ =−1.

The HWT of the input sonogram sono(x,y) is obtained as the scalar product between this image and the hypercomplex MW (Equation (2)):

(2) $$\mathrm{HWT}\left\{\mathrm{sono}\left(x,y\right)\right\}=\mathrm{sono}\left(x,y\right),\psi_{a}\left(x,y\right)$$

During image acquisition, the acquired data are often corrupted by noise. The noise randomly affects the intensity values of each pixel in a noisy image. The aim of the proposed algorithm is to reduce the noise level while preserving the main features of the image. The multiplicative noise component (sono = sono_useful_ × sono_noise_) is transformed into an additive one through logarithm computation (Equation (3)):

(3) $$\log\;\mathrm{sono}=\log{\mathrm{sono}}_{\mathrm{useful}}+\log{\mathrm{sono}}_{\mathrm{noise}}\;$$

Because this new operator is somewhat polarized, we also conceived a mean correction procedure. Next, the HWT is used for decomposing the image affected by additive noise on different levels, so as to check different frequencies (layers) of the same input signal at each level applied the ASTF. The advantages of using wavelets are the improvements of the processing speed and the decorrelation of the noise wavelet coefficients.

We propose an ASTF for filtering, which is a soft thresholding filter whose threshold is adaptively selected in moving windows in the function of the noise level. Another parameter is the current standard deviation of the useful image. This filter is suited for additive noise applied in the wavelet domain [22,23]. The filter is adaptive, taking into account the characteristics of the input image (the maximal wavelet coefficient value, which is different for each acquired image). There are two parameters of the ASTF that need to be estimated, namely σ_noise_ and σ_useful._ As realistic assumption, based on previous studies [24,25], the noise distribution is assumed to be a zero mean Gaussian one with the standard deviation σ_noise_, while the useful distribution is tailored as a Laplacian repartition with the standard deviation σ_useful_. The input–output relation of the ASTF filter to the wavelet coefficients of the sonographic image sono:

(4) $${\mathrm{filter}}_{\mathrm{sono}}=\left\{\begin{array}{ccc} \mathrm{sono}+T_{\mathrm{sono}}, & & \mathrm{sono}<-T_{\mathrm{sono}}\\ 0, & T_{\mathrm{sono}} & <\mathrm{sono}<T_{\mathrm{sono}}\\ \mathrm{sono}-T_{\mathrm{sono}}, & & \mathrm{sono}>T_{\mathrm{sono}} \end{array}\right.$$

The algorithm is personalized and tailored to the characteristics of each scanned neonate, as no fixed threshold value is applied. The threshold T_sono_ is computed with the following relation:

(5) $$T_{\mathrm{sono}}=\sqrt{2}\frac{{\hat{\sigma }}_{\mathrm{noise}}^{2}{{}_{}^{}}_{}^{}}{{\hat{\sigma }}_{\mathrm{useful}}}.$$

where the noisy and useful components depend on the wavelet coefficients (specific for each image) and on the parameters computed for each scanned image:

(6) $${\hat{\sigma }}_{\mathrm{noise}}=\frac{\mathrm{median}\left(\left|y\right|\right)}{0.6745},\;y\in D_{1}^{3}$$

## 3. Results

Noisy recordings of two male and one female patient have been chosen to exemplify the performance of the proposed HWT filtering procedures. HWT has been applied on four levels of decomposition, using the Daubechies orthogonal mother wavelet with a number of two vanishing moments (for a better time–frequency localization). For the noisy echographic image two clinical aspects (a general view of the scanned area and a zoom of area of interest) are observed.

**Figure 5:**
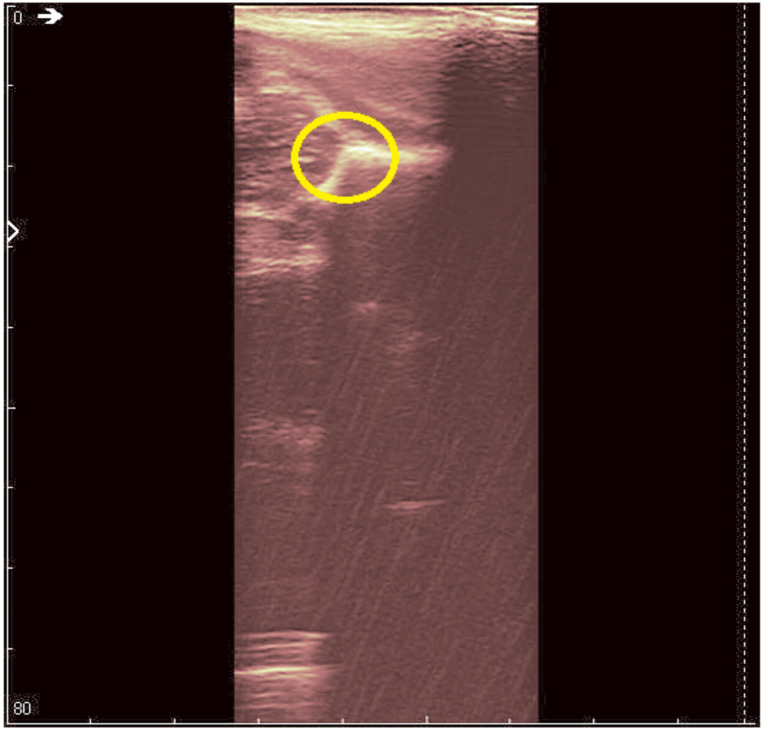
Same region of interest as in Figure 4 was encircled in yellow. The interior speckle grains have disappeared.

The original recording is displayed in Figure 3. As a first processing step, a color palette has been added to the original ultrasound in order to better highlight the noise and noise-free zones (Figure 4). The wavelet-based filter, as described in Section 2, is applied, and the processed image is shown in Figure 5.

**Figure 6:**
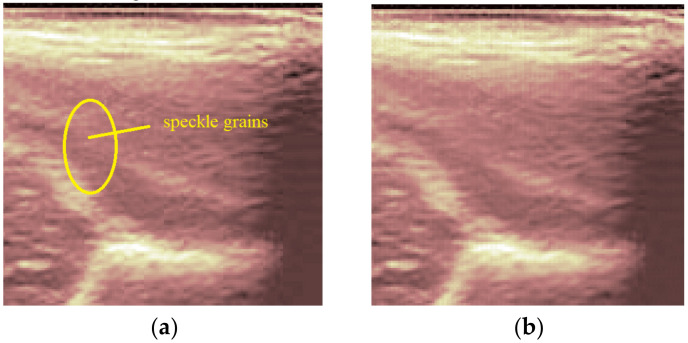
Comparison between the same zoomed region of interest in the original image (**a**) and the denoising result (**b**).

We can remark, when analyzing the zoomed parts of Figure 4 and Figure 5 (the acetabulum), that in the original image speckle noise appears, strongly affecting the image. The filtering system proposed in this paper does not completely remove the speckle but brightens the image, at the same time not oversmoothing the original image. The main bone structure can be better outlined. A comparison between the same zoomed region of interest in the original image and denoising result, showing the disappearance of the speckle grains (Figure 6)—resulting thus in a smoother image where anatomical features can be better outlined.

**Figure 7:**
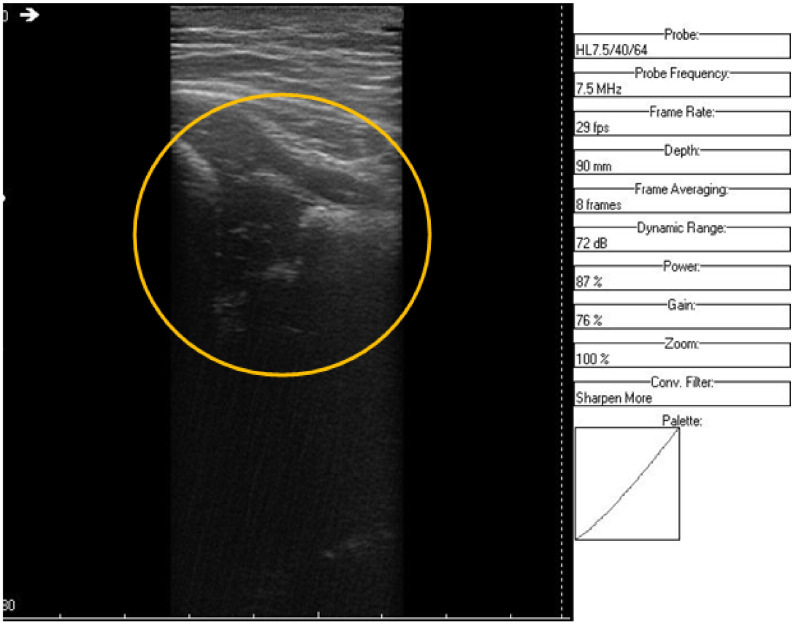
Original data, Image 2.

**Figure 8:**
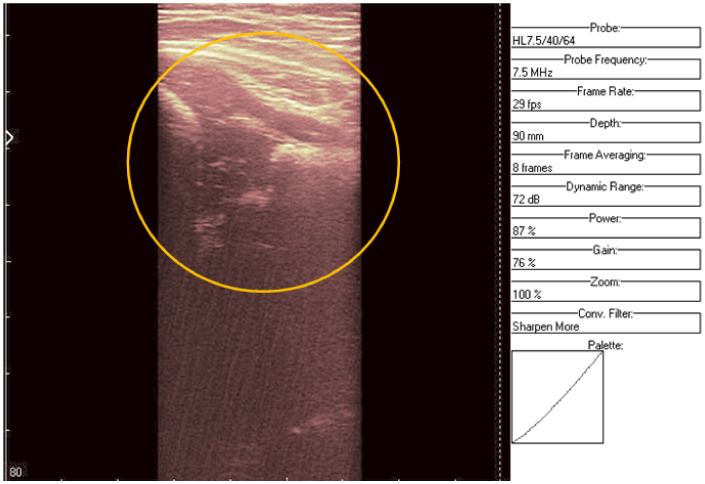
Original data, Image 2, with added color palette and area of interest zoomed.

**Figure 9:**
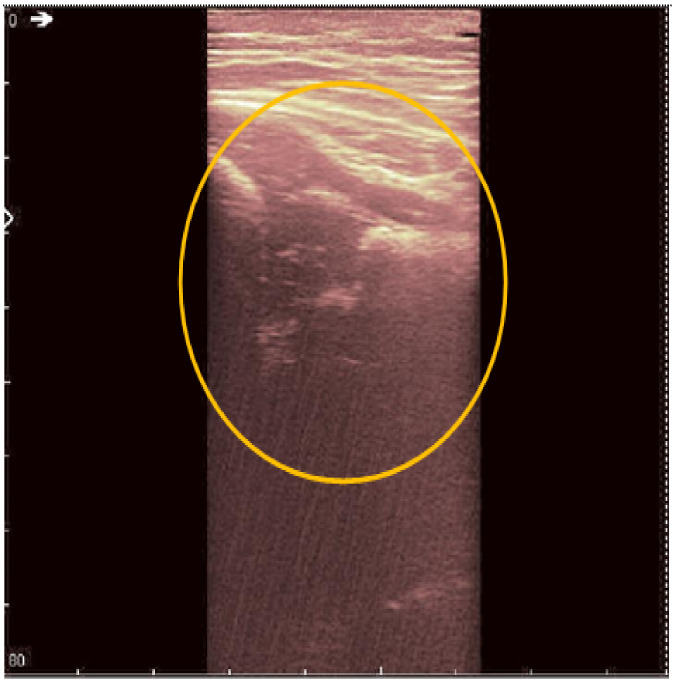
Denoising result for Image 2.

The second image (Figure 7 and Figure 8) is a hip socket sample taken from a female patient. Not many details can be noticed in the original image. The result of the proposed denoising system is presented in Figure 9. The anatomical structures can be observed more clearly and more detailed than before. In this study, we aimed not to debate the medical diagnoses, but to design an algorithm which can provide images at a better resolution, so as to help both the physician and the child patient.

**Figure 10:**
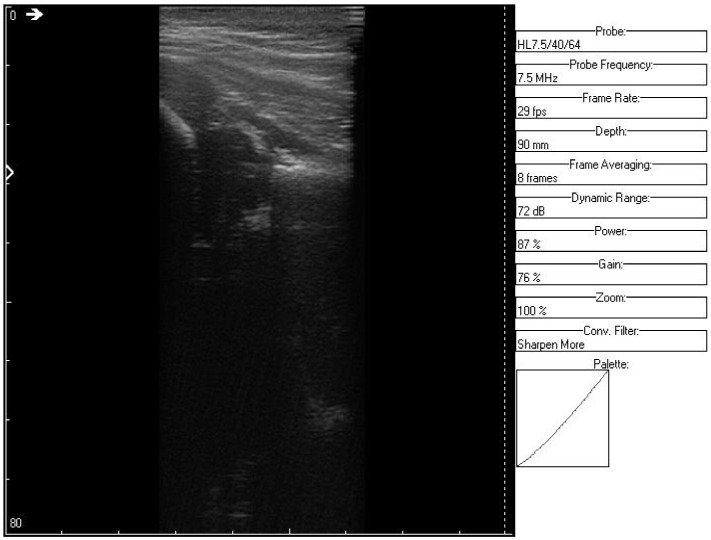
Original data, Image 3.

**Figure 11:**
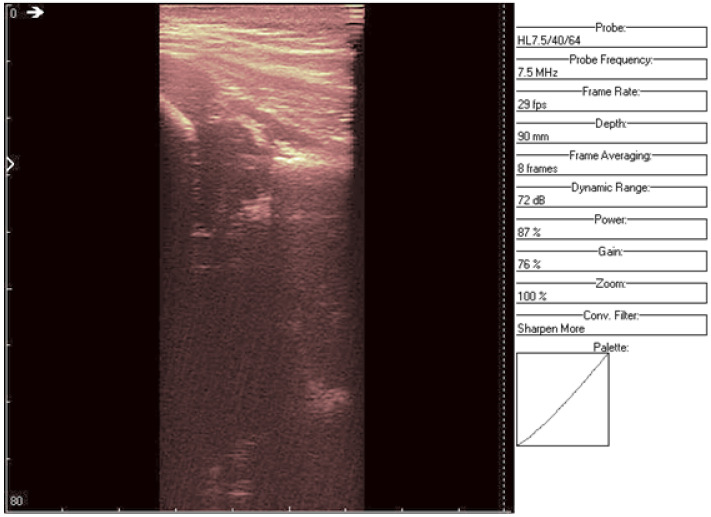
Original data, Image 3, with added color palette.

An ultrasound (Image 3 (Figure 10 and Figure 11)) was taken of a male patient with a sample frequency of 7.5 MHz and a frame rate of 29 fps. The parameters are same as in the previous two cases, yet the image of the acetabulum is hardly visible.

**Figure 12:**
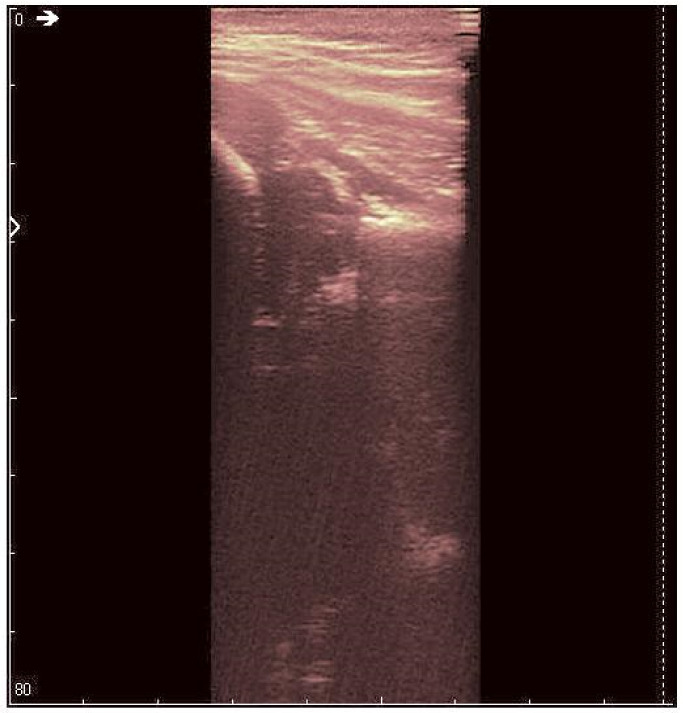
Denoising result for Image 3.

After applying the HWT filtering (Figure 12), the acetabulum, the femoral head, and the femur can be outlined by any physician.

We may conclude, when comparing last two images, that after filtering several details can be seen, easing the process of diagnosis for a trained physician. The denoised images are brighter and more details (not visible on the original image) can be distinguished.

To improve the analysis of the results we add the values of some quality measurements as well. One of the most used quality measurements for the evaluation of an image processing method is the Peak Signal to Noise Ratio (PSNR), defined as:

(7) $$\mathrm{PSNR}=20\log_{10}\frac{255}{\sqrt{\sum_{k=1}^{p}\sum_{l=1}^{q}{\left(f\left(k,l\right)-g\left(k,l\right)\right)}^{2}}},$$

where *f* represents the input image, *g* represents the denoised image, *p* is the number of lines, and *q* the number of columns of that images. Thus, for the three cases of application of the proposed denoising method we obtained the PSNRs in Table 1:

These high PSNR values indicate that the proposed denoising method is indeed optimal, realizing a very good treatment of homogeneous regions. The best result was obtained in the third experiment and the poorest result in the case of the second experiment. Usually, performance is related to the noise level contained in the processed image (dealing with children, the noise level is highly correlated with their ability to manage the ultrasound recording procedure). Nevertheless, the PSNRs are quite high in all cases.

## 4. Discussion

The goal of the study was to reduce exposition time for neonates during necessary procedures, such as hip ultrasounds, and ease the obtainment of a diagnosis. Noiseless images are seldom encountered in practice, as a child is naturally moving constantly, thus adding unwanted echoes. This type of noise is named speckle and has a multiplicative nature. We propose a homomorphic filtering method based on the HWT, transforming multiplicative noise into additive noise through logarithm computation. Additionally, an adaptive ASFT filter is applied, with a threshold tailored to suit the neonate’s specific structure. The algorithm is thus personalized, searching for the optimal results of each recorded echogram. The ASTF’s optimality is due to the equivalence of this type of filter with the LASSO regulator. We selected a filtering method applied in the wavelet domain because wavelets have some advantages, such as a good time–frequency localization, the speed of the WT algorithm, and the ability to decorrelate the noise. In particular, the HWT has some noticeable advantages over other WTs, including the quasi shift invariance, the better directional selectivity, and the ability for hyperanalysis.

We evaluated the proposed approach on real images, acquired in Timisoara. Through filtering, more accurate images were obtained, displaying more clearly the areas of interest (acetabulum, angles to be measured). The denoising method proposed rejects a part of the speckle noise and as a consequence it increases the contrast of the image. Both effects facilitate the visual analysis of the echogram. This contrast enhancement effect can be improved by making a non-linear transformation of the magnitudes of the HWT coefficients after the use of the ASTF [26]. The ultrasound check can therefore be carried out more quickly for neonates and the diagnosis can be more quickly obtained.

Future research directions envisage the checking of the algorithm’s performances with other mother wavelets (Symmlet, Coiflet) or even a new family of mother wavelets, such as the biorthogonal mother wavelets, which use different filters for the decomposition and reconstruction of the signal.

## 5. Conclusions

The goal of this paper is to propose an HWT-based filtering algorithm for neonate ultrasound images. The HWT transform is flexible and quasi-invariant to translation, with good directional selectivity. We performed a simple implementation of this transform, based on the reduction to the input principle. Any orthogonal or biorthogonal real mother wavelet can be used for the computation of HWT.

As a first pre-processing step, we added a palette color to the original image to enlighten the areas of interest. Our goal is to make ultrasound images easier to interpret and diagnose, so the time necessary for acquisition is reduced (a benefit for the neonate patient). Additionally, the images need to be reliable, and a filtering method has been established to remove the main artifact echoes (due to involuntary movements or the breathing of the patient). After filtering, several details can be noticed, easing the process of diagnosis for a trained physician. The images are brighter and more details (not seen on the original image) can be distinguished after processing. We also computed the PSNRs of the three denoising results obtained and we obtained high values, objectively showing the high quality of the denoising method proposed.

Future studies will aim further than the validation of the processing method and will be applied to larger populations of neonates. It is possible to find a mother wavelet other than the Daubechies mother wavelets with the two vanishing moments used in this study, which improved the PSNR of the medical ultrasound images of the hip. Any medical device which eases medical diagnosis, especially if difficult situations arise, such as over-fatigue or lack of sufficiently trained personnel [27], is of practical clinical use.

## Figures and Tables

**Table 1:** The PSNR values obtained for each studied image.

Number of Experiment (Image)	First Image	Second Image	Third Image
PSNR	49.1 dB	47.9 dB	49.38 dB

## Data Availability

Not applicable.
